# Ascites, a New Cause for Bilateral Hydronephrosis: Case Report

**DOI:** 10.1100/tsw.2009.112

**Published:** 2009-10-02

**Authors:** Deepika Jain, Smrita Dorairajan, Madhukar Misra

**Affiliations:** Department of Internal Medicine-Nephrology, University of Missouri, Harry S Truman VA Hospital, Columbia, USA

**Keywords:** cirrhosis, hydronephrosis, obstructive uropathy, acute renal failure

## Abstract

Bilateral hydronephrosis secondary to urinary obstruction leads to a buildup of back pressure in the urinary tract and may lead to impairment of renal function. We present a case of a 57-year-old male with a history of alcoholic liver cirrhosis, who presented with tense ascites and acute renal failure. Bilateral hydronephrosis was seen on abdominal ultrasound. Multiple large-volume paracenteses resulted in resolution of hydronephrosis and prompt improvement in renal function.

## INTRODUCTION

The etiology of urinary tract obstruction varies among the population, depending on age, sex, and other concurrent medical conditions. Such obstruction can also be classified based on duration, site, or age of onset. The incidence is highest in the first decade of life, primarily as a result of congenital anomalies of the urinary tract. A second peak is witnessed after the sixth decade of life, most commonly resulting from obstructive disorders of the prostate. Congenital conditions, such as posterior urethral valves or bladder diverticulae, can cause bilateral hydronephrosis in the early years. In the later years, extrinsic compression leading to bilateral hydronephrosis may be caused by pregnancy, pelvic organ prolapse, pelvic malignancy, prostate cancer, or hyperplasia[1], in addition to other causes.

We report the case of a patient who presented with tense ascites associated with acute renal failure, who was found to have bilateral hydronephrosis. Resolution of hydronephrosis was seen following multiple paracenteses and was accompanied by improvement in renal function.

## CASE REPORT

A 57-year-old male was admitted to our hospital with worsening ascites and acute renal failure. One year ago, he was diagnosed with decompensated cirrhosis of the liver secondary to hepatitis C and alcohol abuse, with associated ascites, portal hypertension, and esophageal varices. On presentation, the patient had tense ascites and acute renal failure, with blood urea nitrogen of 60 mg/dl (normal: 8–26) and creatinine of 2.84 mg/dl (normal: 0.61–1.28). Blood urea nitrogen was 10 mg/dl and serum creatinine was 1.2 mg/dl approximately 3 months prior to presentation. His home medications included furosemide 120

mg/day, spironolactone 300 mg/day, and propanolol 10 mg twice a day. The patient was asymptomatic at the time of admission except for abdominal distension. He denied any recent changes in the medications or any urinary symptoms. On admission, his blood pressure was 100/71, heart rate 62, regular. He was afebrile. Clinical examination revealed mild bilateral pitting pedal edema. The abdomen was distended, but nontender on exam. No abdominal mass was palpable. Fluid thrill was present. Examination of other systems was unremarkable. Urine analysis was normal. His BUN was 60 mg/dl and serum creatinine was 2.81 mg/dl. The fractional excretion of sodium was measured and was 0.46%, suggestive of prerenal etiology of ARF. Henceforth, aggressive volume resuscitation was tried initially, along with careful monitoring of intake and output. There was minimal improvement in the renal function. Ultrasonography of the abdomen showed massive ascites, and mild to moderate hydronephrosis, which was not documented on an ultrasound exam performed 6 months ago. A CT scan of the abdomen, without intravenous contrast, was done to further delineate the site of obstruction. No abdominal mass or calculi were seen. Following this, a total of about 6 l of ascitic fluid was drained over a period of 5 days. The fluid on biochemical analysis showed that total cell count was 23/mm$^3$ of which eight were white blood cells and the remaining were red blood cells. The ascitic fluid albumin was less than 0.6 gm/dl, glucose was 123 mg/dl, LDH was 33 U/l, and amylase was less than 27 U/l. The serum level of glucose was 123 mg/dl, total protein was 5.5 gm/dl, and albumin was 1.6 gm/dl. The serum ascitic albumin gradient (SAAG) was calculated to be 1.0. The renal parameters were monitored daily and showed steady improvement (creatinine was 1.82 mg/dl by day 6). Table 1 summarizes the daily renal function and the volume removed through paracentesis. Repeat ultrasonography of the abdomen was done which showed interval resolution of bilateral hydronephrosis.

**TABLE 1**
**Daily Renal Function and Volume Removed**

| Date | Serum Creatinine (mg/dl) | Urine Volume (ml in 24 h) | Paracentesis (Yes/No) | Volume Removed (ml) |
|---|---|---|---|---|
| 3/13/09 | 2.81 | 550 | Yes | 40 (diagnostic) |
| 3/14/09 | 2.68 | 700 | No | NA |
| 3/15/09 | 2.72 | 690 | No | NA |
| 3/16/09 | 2.73 | 2080 | Yes | 1250 |
| 3/17/09 | 2.39 | 1225 | No | NA |
| 3/18/09 | 2.31 | 1025 | Yes | 4500 |
| 3/19/09 | 1.82 | 1475 | No | NA |

Figs. 1–4 show the hydronephrosis in both right and left kidneys, and the resolution of the same after paracentesis.

## DISCUSSION

There are many causes of obstructive uropathy, such as bladder outlet obstruction, posterior urethral valves, or extrinsic causes, such as pregnancy and pelvic malignancy. The causes can be congenital or acquired. Congenital anomalies may obstruct the urinary tract at any level from the ureteropelvic junction to the tip of the urethra. Even though some of these lesions occur rarely, these represent an important cause of urinary tract obstruction because in younger patients, they can lead to severe renal impairment and may result in end-stage renal disease. The acquired causes can be extrinsic or intrinsic. Among intrinsic causes, nephrolithiasis represents a common cause of obstruction in young males, while extrinsic acquired causes

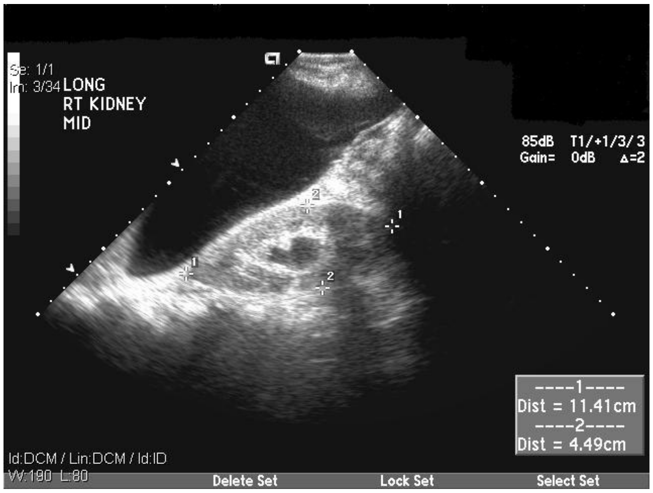

**FIGURE 1**. USG abdomen shows right kidney with hydronephrosis.

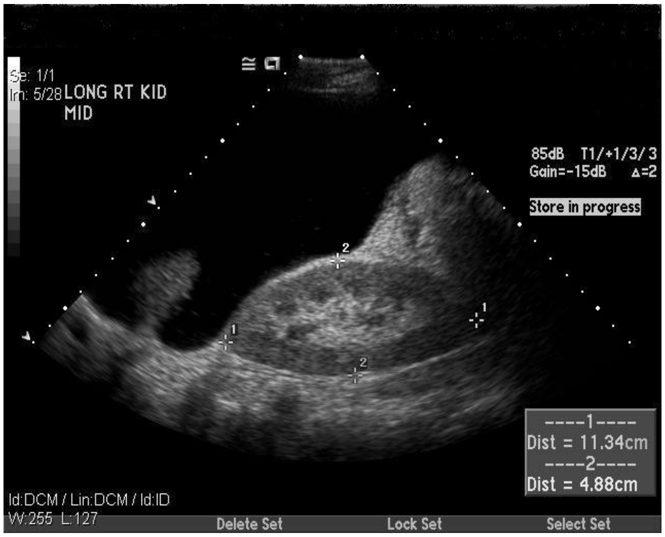

**FIGURE 2.** USG abdomen shows right kidney with interval resolution of hydronephrosis.

are seen in a wide variety of settings. These causes are more common in younger females due to the relatively higher frequency of obstructive uropathy resulting from processes in the female reproductive tract, such as pregnancy and pelvic neoplasms. After the age 60, it becomes relatively more common in males, most frequently due to benign prostatic hyperplasia causing outlet obstruction.

In cirrhosis of the liver, the renal impairment can be multifactorial. Common causes are prerenal secondary to overdiuresis or extravascular spacing of fluids, leading to decreased effective circulating volume. Hepatorenal syndrome is a clinical condition described in such patients caused by severe vasoconstriction of renal circulation. It results from extreme underfilling of arterial circulation in patients with advanced cirrhosis. Apart from the usual clinical presentation of elevated creatinine, which does not improve with plasma expansion trial and normal urine analysis with absent proteins or blood, the renal imaging is normal with no evidence of obstructive uropathy or renal parenchymal disease. It is thus a diagnosis of exclusion.

Since the renal function improved rapidly with relief of ascites in our patient, the likelihood of hepatorenal etiology was less, as in this syndrome the renal function does not improve rapidly.

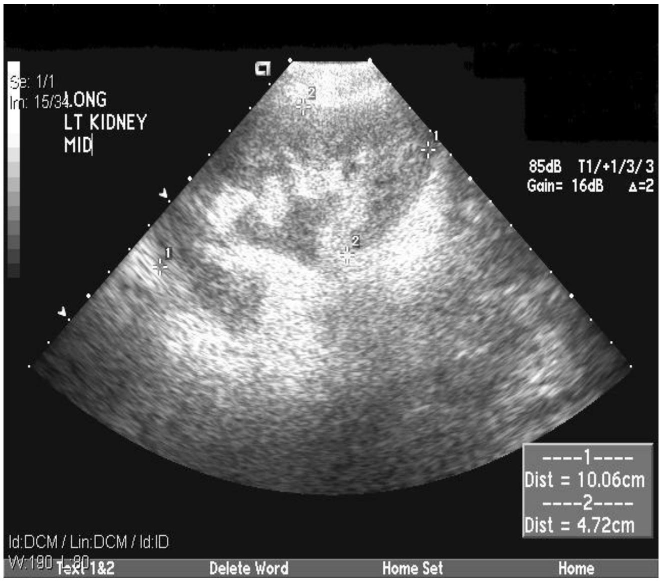

**FIGURE 3.** USG abdomen shows left kidney with hydronephrosis.

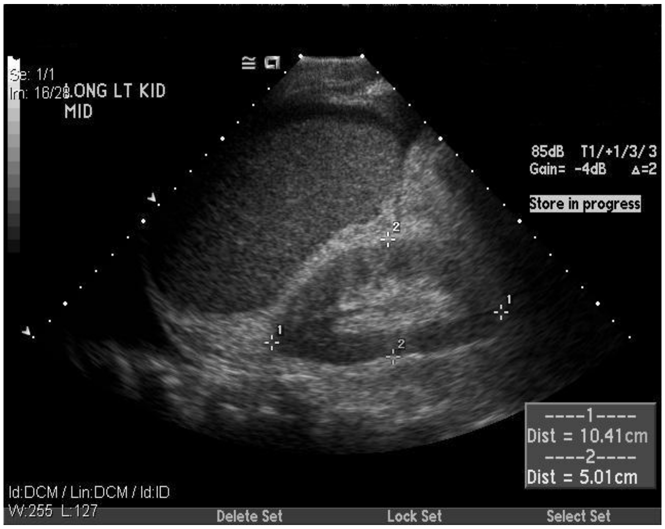

**FIGURE 4.** USG abdomen showing interval resolution of hydronephrosis in left kidney.

We hypothesized that high pressures in the abdomen could have led to a functional obstruction and hydronephrosis. In his review on abdominal compartment syndrome, Paula[2] described that cirrhosis leading to tense ascites can sometimes cause chronic intra-abdominal hypertension. In our patient, described above, bladder pressures were measured to assess the intra-abdominal pressures. The pressures were mildly elevated, ranging between 26 and 30 mmHg.

In our review of the literature, we found only one reported case, thus far, where ascites was considered to be the cause of the hydronephrosis. Calabia de Diego et al.[3] described a 30-year-old female who developed acute renal insufficiency with massive ascites following liver transplantation. In their case, imaging showed pelvic floor organ prolapse. To evaluate the etiology further, they did helical CT of the abdomen and found that pelvic organ prolapse was secondary to massive ascites, and there was no uterine compression to the ureters. Since genital prolapse is a well-known cause of hydronephrosis, they ruled it out with the CT scan of the abdomen, thus suggesting ascites as the cause of hydronephrosis.

There have been cases mentioned in the literature where giant hydronephrosis presented as ascites. Singh et al.[4] reported a case where a patient presented with massive ascites. On surgical exploration of the abdomen, he was found to have giant hydronephrosis of the right kidney. About 20 l of hemorrhagic fluid was removed from the right kidney. A similar case has been reported by Ilter et al.[5].

We report a case of ascites causing functional obstruction leading to hydronephrosis that resolved on paracentesis. Ascites can therefore be added as one other cause of bilateral hydronephrosis, a rare but possible etiology.
